# Evaluation of Bio-VOC Sampler for Analysis of Volatile Organic Compounds in Exhaled Breath

**DOI:** 10.3390/metabo4040879

**Published:** 2014-09-29

**Authors:** Jae Kwak, Maomian Fan, Sean W. Harshman, Catherine E. Garrison, Victoria L. Dershem, Jeffrey B. Phillips, Claude C. Grigsby, Darrin K. Ott

**Affiliations:** 1 The Henry M. Jackson Foundation for the Advancement of Military Medicine, Air Force Research Laboratory, 711th Human Performance Wing/RHXBC, Wright-Patterson AFB, OH 45433, USA; E-Mails: cegarris@mit.edu (C.E.G.); victoria_dershem@mymail.eku.edu (V.L.D.); 2 Air Force Research Laboratory, 711th Human Performance Wing/RHXBC, Wright-Patterson AFB, OH 45433, USA; E-Mails: maomian.fan@us.af.mil (M.F.); claude.grigsby@us.af.mil (C.C.G.); 3 UES, Air Force Research Laboratory, 711th Human Performance Wing/RHXBC, Wright-Patterson AFB, OH 45433, USA; E-Mail: sean.harshman.ctr@us.af.mil; 4 Naval Medical Research Unit—Dayton, Wright-Patterson AFB, OH 45433, USA; E-Mail: jeffrey.phillips.17@us.af.mil; 5 Air Force Research Laboratory, 711th Human Performance Wing, U.S. Air Force School of Aerospace Medicine Wright-Patterson AFB, OH 45433, USA; E-Mail: darrin.ott@us.af.mil

**Keywords:** Bio-VOC breath sampler, volatile organic compounds (VOCs), thermal desorption, gas chromatography-mass spectrometry (GC-MS)

## Abstract

Monitoring volatile organic compounds (VOCs) from exhaled breath has been used to determine exposures of humans to chemicals. Prior to analysis of VOCs, breath samples are often collected with canisters or bags and concentrated. The Bio-VOC breath sampler, a commercial sampling device, has been recently introduced to the market with growing use. The main advantage for this sampler is to collect the last portion of exhaled breath, which is more likely to represent the air deep in the lungs. However, information about the Bio-VOC sampler is somewhat limited. Therefore, we have thoroughly evaluated the sampler here. We determined the volume of the breath air collected in the sampler was approximately 88 mL. When sampling was repeated multiple times, with the succeeding exhalations applied to a single sorbent tube, we observed linear relationships between the normalized peak intensity and the number of repeated collections with the sampler in many of the breath VOCs detected. No moisture effect was observed on the Tenax sorbent tubes used. However, due to the limitation in the collection volume, the use of the Bio-VOC sampler is recommended only for detection of VOCs present at high concentrations unless repeated collections of breath samples on the sampler are conducted.

## 1. Introduction

Monitoring volatile organic compounds (VOCs) released from exhaled breath has been used as a valuable tool to determine an acute or chronic exposure of humans to chemicals. Gas chromatography-mass spectrometry (GC-MS) is frequently employed to analyze breath VOCs. With recent developments in analytical instrumentation (e.g., proton transfer reaction mass spectrometry (PTR-MS), selected ion flow tube mass spectrometry (SIFT-MS), *etc.*), VOCs can be monitored in real-time so that changes in breath volatile profiles due to short-term events that alter breathing rate and volume (e.g., exercise) may be monitored [1]. Prior to breath analysis for VOCs, breath samples are often collected with canisters or plastic bags and then concentrated with concentrating devices, such as solid phase microextraction (SPME) or sorbent tubes [2]. Recently, the Bio-VOC^TM^ breath sampler, a commercial sampling device, has been introduced and offers several advantages over the above techniques. 

A prototype of the Bio-VOC breath sampler was developed by the Health and Safety Laboratory (Sheffield, UK) [3] and commercialized by Markes International (South Wales, UK). The Bio-VOC breath sampler has three components: a mouthpiece, a volumetric sampler (tube/syringe) that retains around 100 mL air, and a plunger. A subject breathes through a disposable cardboard mouthpiece into the plastic Bio-VOC sampler, which has an open end allowing air to be displaced as exhalation proceeds. As a result, the Bio-VOC sampler allows for the collection of alveolar air, the last portion of the exhaled breath, which is more likely to represent air from deep in the lungs. Once the breath collection is complete, the sampler is capped and VOCs are concentrated using SPME fibers, inserted into the sampler [4,5], or on sorbent tubes, collected by discharging VOCs with a plunger onto tubed media. The latter is most commonly used.

Using the Bio-VOC breath sampler, van den Velde *et al.* [6] compared the differences in VOCs of mouth, the early portion of breath, and alveolar air of 40 subjects. Forty-seven VOCs exhibited significant differences between mouth and alveolar air. Notably, endogenous compounds and/or metabolites of exogenous compounds such as isoprene, acetone, dimethyl sulfide and allyl methyl sulfide were higher in the alveolar air, whereas exogenous compounds such as longifolene, 2-ethyl-1-hexanol, diethyl phthalate, naphthalene, chloroform and hydrocarbons were higher in the mouth air. This study demonstrates that the Bio-VOC sampler can collect, without difficulty, the alveolar air, which contains more endogenous and less exogenous compounds. Additionally, the Bio-VOC device is easy to use with no special training required. Because of these advantages, the Bio-VOC sampler has been routinely used for monitoring occupational and environmental exposure to chemicals [7,8,9,10], VOCs associated with tobacco smoking [11] and halitosis [12], and in the investigation of potential volatile biomarkers for diseases, such as cirrhosis [13,14], chronic obstructive pulmonary disease [15], non-small cell lung cancer [4,16,17], and breast cancer [18]. Additionally, the Bio-VOC device has been used to capture breath gases, such as nitrous oxide (N_2_O) [7,19].

A major limitation of the Bio-VOC sampler is the volume of the air collected. The reported volume for the device ranges from 100 to 150 mL [4,5,6,9,10,15,19,20], which is much lower than the volume collected with other sampling methods. However, no study has successfully measured the actual volume of air collected using the Bio-VOC sampler. To collect the additional volume often required for detection of compounds present at trace levels, many studies repeat the sampling procedure multiple times, collecting breath VOCs on a single sorbent tube [6,10,11,12,13,18,20]. Several problems have been reported with multiple collections using the Bio-VOC breath sampler. Hryniuk and Ross [20] reported that the amount of breath acetone increased proportionally as collection was repeated up to six times. However, they observed no linear response between the amount of isoprene and the number of repeated collections when the sampling procedure was repeated more than three times. The authors suggest this result may be due to the adsorption of water on the multi-component sorbent tubes used (*i.e.*, Tenax and Carbopack B) in the study preventing isoprene adsorption. Since this study only measured the two breath compounds, further validation would be required, by measuring additional compounds, to validate this hypothesis. Scheepers *et al.* [10] reported another problem caused by the adsorption of water on sorbent tubes during the multiple collections (three times), of exhaled breath, with the Bio-VOC sampler. They observed the formation of ice in the cold trap inside the thermal desorber due to the adsorption of water on the Tenax sorbent that was transferred to the trap. As a result, more than 20% of the collected samples (21 out of 101 Tenax tubes) could not be analyzed [10]. However, the adsorption of water on the Tenax sorbent is unusual, as this material has been shown to adsorb water marginally [21]. This known fact about Tenax makes it the sorbent of choice for use in collection of VOCs in humid samples.

While the use of the Bio-VOC sampler is growing, the information about the device is somewhat limited. Therefore, in this study, we measured the volume of the air collected with the sampler, the relationship between the levels of VOCs captured and the number of repeated collections, and the effect of water on the breath VOC analysis.

## 2. Results and Discussion

**Figure 1:**
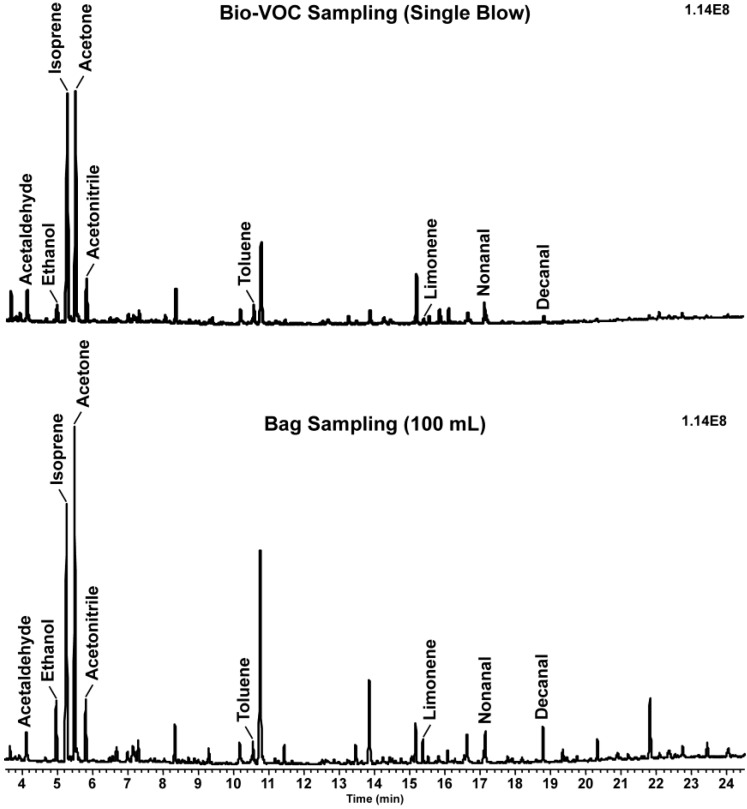
The total ion chromatograms (TICs) of volatile organic compounds (VOCs) detected in exhaled breath samples collected from a male smoker with different sampling methods. Prominent compound peaks include acetaldehyde (4.12 min), ethanol (4.97), isoprene (5.25), acetone (5.48), acetonitrile (5.80), toluene (10.56), limonene (15.53), nonanal (17.14) and decanal (18.79).

The Bio-VOC sampler has a measured water volume of 175 mL. However, the gaseous volume collected could be different due to loss through the opening where displaced air escapes as exhalation proceeds. In order to measure the volume of the air collected in the device, we prepared and analyzed a series of thermal desorption sorbent tubes containing different volumes (50, 100 and 150 mL) of the breath air from a 1 L bag. We created standard curves for the eight major breath VOCs, ethanol, isoprene, acetone, isopropanol, 1-propanol, allyl methyl sulfide, 1-(methylthio)-1-propene, and limonene for each subject (amount of the breath air volume collected from the bag *vs.* intensity ratio of the compound (intensity of the analyte divided by that of the internal standard)). These curves were used to calculate the volume of the breath air collected by a single blow into a Bio-VOC sampler based on the intensity ratio of these compounds. As shown in Table 1, the volume of air calculated ranged between 22 and 153 mL with an average of 88 mL. This large variation in the volumes calculated, is most likely due to different breath sampling methods employed as well as due to different distributions of breath VOCs in each individual. While the Bio-VOC sampler collects the alveolar portion of breath preferentially, bag sampling captures a mixture of air from different parts of the airway even though the subjects were asked to breathe into the bag at the end of a normal resting tidal breath to achieve an alveolar sample. Thus, the use of a bag may not be an ideal approach to determine the volume of breath sample collected with a Bio-VOC sampler. While several methods were carefully considered and attempted to estimate the volume of breath sampled in a Bio-VOC sampler, we concluded that bag sampling was the best method. Using this method, we determined the volume of the breath collected in the Bio-VOC sampler was approximately 88 mL, which is substantially different than the volume measured with water (175 mL). Figure 1 shows the total ion chromatograms (TICs) of the VOCs detected in the exhaled breath of a male smoker using a single Bio-VOC collection (~80 mL) and 100 mL from a bag sample. The data illustrate the Bio-VOC sampler has VOCs with less intensity than the bag sample confirming the volume of the Bio-VOC device is less than 100 mL. These results suggest the Bio-VOC sampler will be best utilized for the analysis of highly abundant compounds when multiple collections are unfeasible.

**Table 1:** The determined volume of breath air collected in a Bio-VOC sampler.

Compound	RT (min)	Quant ion	Volume Calculated (mL)					
			Subject 1	Subject 2	Subject 3	Subject 4	Average	%RSD
Ethanol	4.92	45	52	26	75	102	64	51
Isoprene	5.21	67	85	49	91	148	93	44
Acetone	5.44	43	124	64	116	153	114	32
Isopropanol	5.50	45	58	22	90	81	63	48
1-Propanol	6.61	42	59	73	87	96	79	21
Allyl methyl sulfide	8.99	88	83	62	115	95	89	25
1-(Methylthio)-1-propene	9.76	88	77	69	132	113	97	30
Limonene	15.53	68	88	96	174	75	108	41
Average			78	58	110	108	88	
%RSD			29	43	29	27		

We next determined the relationship between the number of repeated collections with the Bio-VOC sampler and peak intensity ratio of breath VOCs. Unlike the previous finding [20], we observed linear responses in up to five repeated collections with the Bio-VOC sampler for isoprene and many of other breath VOCs detected (Table 2). However, as shown in Table 2, some VOCs do not show a linear relationship. For example, methyl isobutyl ketone shows linear regression with a negative slope and the mean regression coefficient (R^2^) value of 0.1477, suggesting the amount does not change with repeated collections on the Bio-VOC sampler. We believe this compound is likely derived from the room air. However, this is not the case for all exogenous compounds. For instance, toluene and isopropanol show a linear relationship of intensity ratio by the number of repeated Bio-VOC collections with high R^2^ (Table 2), indicating that these exogenous compounds are derived from breath, particularly from the alveolar regions of the lung. Food-derived VOCs such as allyl methyl sulfide and 1-(methylthio)-1-propene, also exhibited a linear relationship (Table 2), suggesting that these dietary compounds are released as a result of gas exchange. Some VOCs (e.g., ethanol, acetophenone, benzaldehyde, phenol, *etc.*) had regression coefficients with large %RSD, indicating that these compounds could be derived from breath for some subjects or from the room air or other exogenous sources for others. Notably, benzaldehyde, acetophenone and phenol are known bleeding compounds derived from Tenax thermal desorption tubes used to concentrate breath VOCs in this study. These results suggest that VOCs derived from the alveolar regions of the lung are preferentially concentrated on a thermal desorption tube with repeated collections of breath sample on the Bio-VOC sampler.

The non-linear response (*i.e.*, saturation) of isoprene during the multiple collections reported by Hryniuk and Ross [20] is likely due to the adsorption of water derived from breath samples on the thermal desorption sorbent tubes and/or the cold trap in the thermal desorber. We have not observed any moisture effect in our analysis with up to five repeated collections using the Bio-VOC sampler and Tenax sorbent tubes. We previously observed a moisture effect on sorbent tubes containing the strong adsorbent carbon molecular sieves (Soil Vapor Intrusion (SVI^TM^) from Perkin Elmer (Waltham, MA, USA)) (Unpublished result). Note that a moisture effect is characterized by a retention time shift in the GC analysis. However, we have not noticed any moisture effect, even with the SVI tubes, during repeated breath collections using the Bio-VOC sampler up to five times (Data not shown). Adsorption of isoprene and acetone on the Bio-VOC device after breath sampling was observed up to 10% of their levels detected in the breath samples, but they can be removed mostly by flushing the sampler with air up to five times (Data not shown).

**Table 2:** Retention time, quantitative ion and regression coefficient (R^2^) for the breath volatile organic compounds identified from repeated collections using the Bio-VOC sampler. R^2^ values are provided to demonstrate linearity of compounds over multiple collections. The list of the compounds was sorted in decreasing order of the mean R^2^.

Compound	RT (min)	Quant ion	R^2^					
			Subject 1	Subject 2	Subject 3	Subject 4	Average	%RSD
Allyl methyl sulfide	8.99	88	0.9878	0.9536	0.9869	0.9947	0.9808	1.88
Toluene	10.53	91	0.9765	0.9534	0.9887	0.9946	0.9783	1.86
1-(Methylthio)-1-propene	9.77	88	0.9855	0.9440	0.9776	0.9814	0.9721	1.96
Isopropanol	5.50	45	0.9510	0.9592	0.9842	0.9697	0.9660	1.48
Acetone	5.44	43	0.9452	0.9433	0.9156	0.9727	0.9442	2.47
p/m-xylene	12.65	91	0.8528	0.9403	0.9539	0.9511	0.9245	5.21
Ethylbenzene	12.49	91	0.8935	0.9538	0.8672	0.9713	0.9215	5.34
Isoprene	5.20	67	0.9743	0.9486	0.9899	0.7554	0.9171	11.90
Limonene	15.53	68	0.9826	0.9349	0.9436	0.7055	0.8917	14.11
Acetonitrile	5.74	41	0.8254	0.9697	0.9411	0.8165	0.8882	8.85
2-Pentanone	9.09	43	0.8057	0.7903	0.9450	0.9962	0.8843	11.54
1-Propanol	6.62	42	0.6637	0.9463	0.9558	0.9326	0.8746	16.11
2-Butanone	7.25	43	0.7646	0.8809	0.8465	0.9739	0.8665	10.00
Octanal	15.36	84	0.8213	0.6678	0.7334	0.8642	0.7717	11.42
Hexanal	11.40	44	0.6247	0.9508	0.6609	0.8473	0.7709	20.05
Heptanal	13.45	70	0.7807	0.7642	0.6838	0.8416	0.7676	8.47
Acetaldehyde	4.07	44	0.1933	0.8472	0.9302	0.9645	0.7338	49.56
Pentanal	9.26	44	0.5148	0.8941	0.6204	0.8940	0.7308	26.46
Benzene	8.29	78	0.9342	0.8118	0.5231	0.6359	0.7263	25.14
Benzaldehyde	15.16	106	0.5510	0.3867	0.9449	0.9007	0.6958	38.97
Nonanal	17.14	57	0.7859	0.4286	0.5822	0.8831	0.6700	30.45
2,3-Butanedione	7.08	43	0.7890	0.8762	0.6227	0.3775	0.6664	32.93
3-Methylfuran	6.94	82	0.0261	0.9447	0.6842	0.9683	0.6558	66.96
Ethanol	4.92	45	0.6121	0.9686	0.8735	0.0810^(−)^	0.6338	62.83
Decanal	18.80	112	0.5344	0.3728	0.4547	0.8883	0.5626	40.35
Phenol	16.07	94	0.5905	0.1354	0.9752	0.4900	0.5478	63.06
Acetophenone	17.08	105	0.5675	0.0732^(−)^	0.5015	0.7822	0.4811	61.78
3-Heptanone	13.12	57	0.0213^(−)^	0.1726	0.8456	0.4561	0.3739	96.92
Methyl Isobutyl Ketone	10.20	100	0.0007^(−)^	0.0925^(−)^	0.111^(−)^	0.3864^(−)^	0.1477	112.59

^(−)^: The slope was negative.

## 3. Experimental Section

### 3.1. Exhaled Breath Collection

Exhaled breath samples were taken from three male volunteers and a female participant. They were healthy without any known lung problems. The subject demographic is summarized in Table 3. Breath sampling was done in a laboratory where no chemicals were stored. Each subject was asked to breathe deeply through a disposable cardboard mouthpiece into a Bio-VOC^TM^ sampler (Markes International, South Wales, UK). Once the breath air collection was completed, the mouthpiece was removed and a plunger was then inserted into the sampler to discharge the collected breath onto a thermal desorption sorbent tube capturing breath VOCs. To collect additional volume, the sampling procedure was repeated up to 5 times with the succeeding exhalations combined onto a single sorbent tube. All procedures were approved by the Institutional Review Board (IRB) in the Naval Medical Research Unit—Dayton at Wright-Patterson Air Force Base.

**Table 3:** Subject demographic.

	Subject 1	Subject 2	Subject 3	Subject 4
Age	44	59	31	21
Sex	Male	Male	Male	Female
Ethnicity	Asian	Asian	Caucasian	Caucasian
Smoking	No	Yes	No	No

### 3.2. Measurement of the Breath Air Volume Collected in Bio-VOC

Each subject was asked to breathe into a 1-liter ALTEF polypropylene bag (Jensen Inert Products, Coral Springs, FL, USA). Then, the breath air was pulled from the bag through a sorbent tube by a Gilian^®^ Gil*A*ir^®^ PLUS (Sensidyne, St. Petersburg, FL, USA) pump at 100 mL/min. Different amounts of volume (50, 100, and 150 mL) were collected on each tube by pulling air from the bag for different amounts of time (0.5, 1, and 1.5 min), respectively. The sorbent tubes containing the breath VOCs were analyzed by a gas chromatograph-mass spectrometer (GC-MS) as described below. Standard curves of the breath VOCs listed in Table 1 were subsequently generated (amount of the breath air volume collected *vs.* intensity ratio of the compound (intensity of the analyte divided by that of the internal standard)). The intensity ratio of the compounds obtained by the Bio-VOC^TM^ sampler (single blow) was then converted into the amount of volume of breath air collected according to the standard curves. Note that different standard curves were created and used for the calculation of the breath volume collected with the Bio-VOC^TM^ sampler for each subject. The breath samples from each subject were collected with a bag and then immediately with a Bio-VOC sampler to minimize variation of breath VOCs caused by smoking, environment, eating, drinking, *etc.*

### 3.3. Sorbent Tubes

Tenax^®^ TA sorbent tubes were used in this study. They were purchased from Markes International (South Wales, UK). All sorbent tubes were stainless steel and were conditioned prior to use per the manufacturer’s instructions.

### 3.4. Analysis of Sorbent Tubes

Each sorbent tube was analyzed by a TD-100 thermal desorber (Markes International, South Wales, UK) coupled to a Trace GC Ultra-ISQ single quadrupole GC-MS (Thermo Scientific, Waltham, MA, USA). The TD-100 parameters are as follows: tube desorption temp.: 310 °C; tube desorption time: 10 min; flow path temp: 160 °C; trap flow: 50 mL/min; pre-trap fire purge time: 1 min; trap low temp: 25 °C; trap high temp: 315 °C for 5 min; trap heating rate: 40 °C/s (MAX). The VOCs in the sorbent tube was split after delivered to the trap (outlet split; at 3.5:1). A 0.8 L cylinder containing gaseous internal standards (bromochloromethane, 1,4-difluorobenzene, chlorobenzene-d_5_, and 4-bromofluorobenzene; 1 ppm each; Linde Gas North America, Stewartsville, NJ, USA) was connected to the TD-100 and 1 μL of the standards were applied to the sampling end of a sorbent tube prior to the desorption of the tube. An Rxi^®^-624Sil MS column (60 m × 0.32 mm ID × 1.80 μm df; Restek, Bellefonte, PA, USA) was used for GC separations containing a mid-polar stationary phase consisting of cyanopropylphenyl and dimethyl polysiloxane. The GC temperature program started at 40 °C for 1 min, and increased at 10 °C/min to 240 °C where the final temperature was held for 20 min. The total GC analysis time was 41 min. Helium carrier gas was used at a constant flow of 2 mL/min. The mass spectrometer was operated in the electron impact ionization mode at 70 eV. The transfer line temperature was 230 °C and the ion source temperature was 275 °C. The mass scan range was 35–300 *m/z* with a scan time of 0.154 s.

## 4. Conclusions

We measured the volume of the breath air collected in the Bio-VOC sampler, which was estimated to be 88 mL of air. For many of the breath VOCs detected, we observed linear relationships between peak intensity ratio of VOCs and number of repeated collections with the Bio-VOC sampler, up to five collections. Notably, VOCs derived from the alveolar regions of the lung are preferentially concentrated on a thermal desorption tube with repeated collections of breath sample on the Bio-VOC sampler. No moisture effect was observed on the Tenax sorbent tubes evaluated. However, due to the limitation in the collection volume, we only recommend the use of the Bio-VOC breath sampler for detection of VOCs present at high concentrations unless repeated collections of breath samples on the Bio-VOC sampler are conducted.
